# NOD mouse dorsal root ganglia display morphological and gene expression defects before and during autoimmune diabetes development

**DOI:** 10.3389/fendo.2023.1176566

**Published:** 2023-06-02

**Authors:** Marta Corral-Pujol, Berta Arpa, Estela Rosell-Mases, Leire Egia-Mendikute, Conchi Mora, Thomas Stratmann, Alex Sanchez, Anna Casanovas, Josep Enric Esquerda, Didac Mauricio, Marta Vives-Pi, Joan Verdaguer

**Affiliations:** ^1^ Immunology Unit, Department of Experimental Medicine, Faculty of Medicine, University of Lleida and IRBLleida, Lleida, Spain; ^2^ Department of Cell Biology, Physiology and Immunology, Faculty of Biology, University of Barcelona, Barcelona, Spain; ^3^ Genetics, Microbiology and Statistics Department, Universitat de Barcelona, Barcelona, Spain; ^4^ Statistics and Bioinformatics Unit, Vall d’Hebron Institut de Recerca, Barcelona, Spain; ^5^ Patologia Neuromuscular Experimental Departament de Medicina Experimental, Facultat de Medicina, Universitat de Lleida and Institut de Recerca Biomèdica de Lleida (IRBLleida), Lleida, Spain; ^6^ Department of Endocrinology and Nutrition, Hospital de la Santa Creu i Sant Pau and Institut d’Investigació Biomèdica Sant Pau (IIB Sant Pau), Barcelona, Spain; ^7^ Faculty of Medicine, Central University of Catalonia, Vic, Spain; ^8^ CIBER of Diabetes and Associated Metabolic Diseases (CIBERDEM), Instituto de Salud Carlos III, Madrid, Spain; ^9^ Immunology Department, Germans Trias i Pujol Research Institute, Badalona, Spain; ^10^ Department of Cellular Biology, Physiology and Immunology, Faculty of Medicine, Autonomous University of Barcelona, Cerdanyola del Vallès, Spain

**Keywords:** type 1 diabetes, dorsal root ganglion, NOD mouse/mice, neurodegeneration, parasympathetic function, neural regulation, autoimmune diseases/disorders

## Abstract

**Introduction:**

During the development of Autoimmune Diabetes (AD) an autoimmune attack against the Peripheral Nervous System occurs. To gain insight into this topic, analyses of Dorsal Root Ganglia (DRG) from Non-Obese Diabetic (NOD) mice were carried out.

**Methods:**

Histopathological analysis by electron and optical microscopy in DRG samples, and mRNA expression analyzes by the microarray technique in DRG and blood leukocyte samples from NOD and C57BL/6 mice were performed.

**Results:**

The results showed the formation of cytoplasmic vacuoles in DRG cells early in life that could be related to a neurodegenerative process. In view of these results, mRNA expression analyses were conducted to determine the cause and/or the molecules involved in this suspected disorder. The results showed that DRG cells from NOD mice have alterations in the transcription of a wide range of genes, which explain the previously observed alterations. In addition, differences in the transcription genes in white blood cells were also detected.

**Discussion:**

Taken together, these results indicate that functional defects are not only seen in beta cells but also in DRG in NOD mice. These results also indicate that these defects are not a consequence of the autoimmune process that takes place in NOD mice and suggest that they may be involved as triggers for its development.

## Introduction

Autoimmune Diabetes (AD) is an autoimmune-mediated process where a chronic inflammation of the islets of Langerhans called insulitis causes β cell destruction. Most of the autoantigens related to Type 1 Diabetes (T1D) including pro-insulin (1), GAD65 (2) and GAD67 (3), tyrosine phosphatase like protein IA-2, ICA69, S100β, HSP60, and IGRP (4) also follow a nervous system expression pattern. In fact, some previous studies have demonstrated that, simultaneously to the pancreatic β-cell attack, there is an autoimmune response against the Peripheral Nervous System (PNS) that regulates the pancreatic islets activity (5–7). Sensory afferent neurons of the dorsal root ganglia (DRG) innervating pancreatic islets are critical components in the onset of prediabetes, promoting islet inflammation thorough altered glucose homeostasis and progressive β cell stress (5). Some studies in Non-Obese Diabetic (NOD) mice suggest that defects in sensory neurons decrease the neurotransmitters secretion into the islets, thus promoting pancreatic beta cell stress and leucocyte infiltration (8).

A major subset of these primary afferent neurons expresses Transient Receptor Potential Vanilloid-1 (TRPV1) (9), a non-specific cation channel involved in sensing thermotrauma (≥44°C), acid (H^+^) and other biochemical factors (10). They also have an efferent function through local release of mediators such as neuropeptides (substance P [sP] and calcitonin gene related peptide [CGRP]) (11). TRPV1^+^ neurons are of known importance in pro-inflammatory reactions (12) and islet infiltrating lymphocytes express receptors for neuropeptides (13). In this regard, it has been previously reported that NOD mice have a functional defect in sensory cells, particularly in those which express the TRPV1 receptor (11). Mutations in TRPV1 have also been related with T1D in humans (14). Moreover, the TRPV1 gene is localized to the Idd4.1 AD susceptibility locus (15), and Razavi, R. et al. have shown that the suppression of neural TRPV1^+^ in NOD mice reduces leucocyte infiltration and AD onset. All these givens suggest that sensory neurons play some role in AD.

The β cell destruction characteristic of AD is mediated by cytokines and other factors released by and expressed on the surface of the immune cells invading the islets, which trigger secondary pathways of cell death in the target β cells (16). One important mechanism by which cytokines contribute to β cell death is *via* endoplasmic reticulum (ER) stress induction that causes *in situ* accumulation of misfolded proteins, and the consequent triggering of the Unfolded Protein Response (UPR). The activation of the UPR pathway leads to the attenuation of global protein translation and up-regulation of ER chaperones, thus increasing the ER folding capacity and the degradation of irreversibly misfolded proteins (17, 18). Cell apoptosis is activated when the UPR fails to solve ER stress. In addition to protein folding, ER is responsible for most of post-translational modifications (PTM) of membrane-bound and secreted proteins. The ER lumen contains the necessary factors to support this function, including molecular chaperones, ATP, an oxidizing environment and millimolar concentrations of calcium (Ca^2+^) (19). PTM defects may contribute to autoimmune disorders, if proteins are modified differently in peripheral tissues than in the thymus (20, 21). In recent years, ER stress and the adaptive UPR have aroused great interest due to their crucial role in the pathogenesis of multiple diseases including neurodegenerative diseases, cancer, and metabolic disorders. ER is also an important organelle for the maintenance of intracellular Ca^2+^ homeostasis, which is necessary for regulating a variety of cellular functions both in the ER lumen and in the cytosol (19), whereas the loss of Ca^2+^ homeostasis can trigger cell apoptosis (22). Some studies have shown that sensory neurons in the DRGs can be susceptible to ER stress due to their high metabolic activity and protein synthesis demand (23). This can lead to activation of the UPR in an attempt to restore ER function and protect against cellular damage. However, if ER stress persists and is not resolved, it can lead to chronic activation of the UPR and contribute to neuronal dysfunction and damage in DRGs. In the context of diabetes, prolonged ER stress and UPR activation in DRGs have been involved in the development of diabetic peripheral neuropathy, which is a common complication of diabetes characterized by sensory dysfunction and nerve damage in the peripheral nervous system, including DRGs (24).

To delve into this topic, we carried out histopathological and gene expression analyses in DRG sensory neurons from NOD in the pre-diabetic period, and compared them with those from NOD.RAG-2^-/-^ and C57BL/6 mice. The results indicated the existence of morphological and functional abnormalities in the sensory nerve cells of DRG throughout the development of the disease. In view of these results, the study of the expression of a selected group of genes was extended to blood leukocytes, which also showed transcription defects.

## Materials and methods

### Mice

NOD, NOD.RAG-2^-/-^, and C57BL/6 mice were maintained by brother-sister mating under specific pathogen-free conditions at the University of Lleida. In the NOD mouse model, the preclinical stage or insulitis begins at 3 to 6 weeks of age. This progresses over time and promotes the destruction of pancreatic β-cells, so that at 12-14 weeks of life the first cases of insulin-dependent diabetes appear. In our facility, the AD incidence in NOD mice reaches 86% in females and 50% in males. This incidence stabilizes around 32 weeks of age, and thus we considered this as the end point to analyze the presence of vacuoles in non-diabetic mice. Unless otherwise specified, all mice used in this study were pre-diabetic or non-diabetic mice.

This study was carried out in accordance with the principles of the Basel Declaration and recommendations of the Catalan Government (*Generalitat de Catalunya*) concerning the protection of animals for experimentation. The protocol was approved by the Committee on the Ethics of Research in Animal Experimentation of the University of Lleida, Spain. Protocol #: CEEA 02-04/16.

### Ganglia isolation

C57BL/6, NOD and NOD.RAG-2^-/-^ female mice of different ages (two, six, nine, 12, 26 and 32 weeks) were anesthetized with an intraperitoneal injection of sodium pentobarbital and perfused with physiological saline solution, followed by 4% paraformaldehyde (PFA) in 0.1 M phosphate buffer (PB) at pH 7.4. Next, Dorsal Root Ganglia (DRG) were carefully removed and maintained in 4% paraformaldehyde (PFA) at 4°C until their use. DRG mRNA samples from three and 12 weeks old C57BL/6, NOD and NOD.RAG-2^-/-^ female mice were used for the microarray and RT-qPCR assays. The RT-qPCR assays were also carried out with cDNA samples of white blood cells from three weeks old mice of the same strains.

### White blood cells isolation

White blood cells of three weeks old NOD, and C57BL/6 female mice were obtained by a heart puncture with a 25G needle to obtain the blood sample. Erythrocytes were lysed by osmotic pressure.

### RNA isolation

RNA from isolated ganglia was obtained with the FFPE RNA Purification Kit (25300, Norgen Biotek Corp) and transcribed to cDNA with the High-Capacity cDNA Reverse Transcription Kit (4374966, Applied Biosystems).

### Histology

Some ganglia were harvested and fixed with 4% PFA and then included in paraffin blocks. 12 µm sections were stained with routine hematoxylin and eosin (H&E) protocol.

### Electronic microscopy

Some ganglia were harvested and fixed with 4% PFA and then fixed with 2.5% glutaraldehyde in 0.1M phosphate buffer (pH 7.4) for 24 hours. Samples were then incubated in 1% osmium tetroxide for two hours and finally included in Embed 812 medium (Electron Microscopy Sciences, Fort Washington, PA). Semi-thin sections (1μm thick) were stained with toluidine blue and ultra-thin sections of selected areas were stained with uranyl acetate and lead citrate for electronic microscopy analysis.

### Microarray assay

Total RNA concentrations from mouse samples were measured with a Nanodrop 1000 Spectrophotometer (ThermoFisher) and RNA integrity was assessed using the Agilent 2100 BioAnalyzer (Agilent Technologies, USA). All samples showed similar RNA integrity numbers. The Genechip**^©^** Mouse Clariom S 24x arrays plate (ThermoFisher) was used to analyze gene expression patterns on a whole-genome scale on a single array. Starting material was 2 ng of total RNA of each sample. Briefly, sense ssDNA was generated from total RNA with the GeneChip WT Pico Reagent Kit (Thermofisher) according to the manufacturer’s instructions. Then, sense ssDNA was fragmented, labelled and hybridized to the arrays with the GeneChip WT Terminal Labeling and Hybridization Kit (Affymetrix). Arrays plate was scanned and processed with Affymetrix GeneChip Command Console to obtain expression array intensity.CEL files.

### RT-qPCR assay

For Real-Time quantitative PCR (RT-qPCR) the TaqMan Universal Master Mix II, no UNG (4440040, Applied Biosystems) and different TaqMan Gene Expression Assays (4448892, Applied Biosystems) targeting *Ahcy* (Mm01621912_s1), *Arl8a* (Mm01293357_gH), *Rps12* (Mm03030276_g1), *Scg5* (Mm00486077_m1) and *Vcp* (Mm01702786_gH) were used. *GADPH* (4351370, Mm99999915_g1, Applied Biosystems) was used as a constitutive control gene (housekeeping gene).

### Data analysis

A Transcriptome analysis of DRG cells from C57BL/6, NOD and NOD.RAG-2^-/-^ female mice was performed comparing data from mouse strain samples at 3 and 12 weeks of age, and within the same strain regarding the time effect (three *vs* 12 weeks old mice). A cluster analysis of gene expression for all genes selected in one or other comparison was performed to detect expression differences between groups. A hierarchical clustering with correlation distance between genes and eventually euclidean distance between samples was done, although the latter is often omitted to facilitate visualization. For each clustering of selected genes and samples, a heatmap was drawn.

The selection of differentially expressed genes (DEGs) was performed using a linear model for each gene followed by variance regularization based on empirical Bayes modelling as implemented in the R/Bioconductor limma package (25). To deal with the multiple testing issues derived from the fact that many tests (one per gene) were performed simultaneously, p-values were adjusted to obtain strong control over the false discovery rate using the Benjamini and Hochberg method.

To find genes affected by two or more conditions, we performed a multiple comparisons analysis, which allowed to know the number of DEGs common to each pair or three comparisons.

Different bioinformatic tools were used to analyze the microarray results. Those genes with an adjusted p-value<0.05 and a |LogFC|>1 were chosen as genes of interest and information about their function, cellular location and tissue expression was studied.

Gene database (http://www.ncbi.nlm.nih.gov/gene), Mouse Genome Informatics or MGI (http://www.informatics.jax.org/) and GO (http://geneontology.org/) were used to search information about the molecular function and the biological process of the genes and proteins of interest. T1Dbase (http://www.t1dbase.org/) was used to find those significant genes associated with a locus of susceptibility to T1D. Basic Local Alignment Search Tool or BLAST (http://blast.ncbi.nlm.nih.gov/BlastAlign.cgi) was used to analyze the percentage of similarity of the mice candidate genes with their orthologous in humans. WebGESTALT or WEB-based Gene SeT AnaLysis Toolkit (http://www.webgestalt.org) and PantherDB or Protein ANalysis THrough Evolutionary Relationships DataBase (http://www.pantherdb.org) were used to identify any enriched gene category (regarding molecular function, biological process, and cellular component) on our list of DEGs.

### Statistical analysis

Statistical analyses were performed using the Mann-Whitney U test.

## Results

### DRG cells show histological alterations before and during the development of autoimmune diabetes

**Figure 1:**
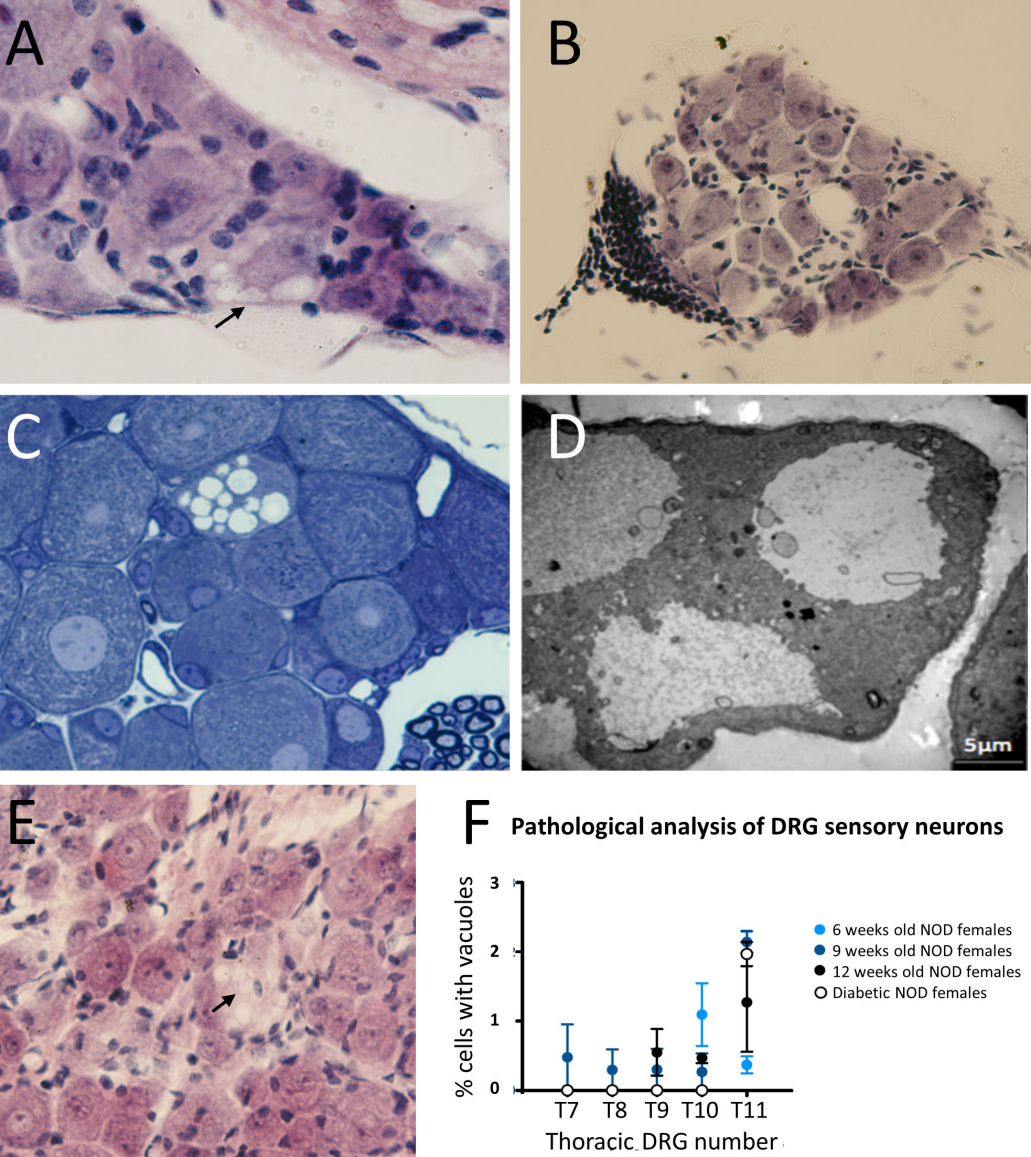
Histological analysis of DRG cells from NOD mice. **(A)** DRG section stained with H&E. Cytoplasmic vacuoles (arrow) are observed in sensory neurons of a 32 weeks old non diabetic NOD female (60X); **(B)** DRG section stained with H&E. Leukocyte infiltration at the DRG periphery of a 26 weeks old non diabetic NOD mouse (20X); **(C)** Semi-thin section (1μm thick) stained with toluidine blue of DRG cells of a 9 weeks old NOD.Rag2^-/-^ female; **(D)** Analysis by electron microscopy of cytoplasmic vacuoles of DRG cells of a 9 weeks old NOD.Rag2^-/-^ female. Vacuole single-membrane is clearly observable; **(E)** DRG sections stained with H&E. Cytoplasmic vacuoles (arrow) in DRG cells of 2 weeks old NOD.Rag2^-/-^ female (40X); **(F)** Percentage of sensory neurons with cytoplasmic vacuoles at T7 to T11 DRGs from 6, 9 and 12 weeks old and NOD diabetic females (n=3).

Sensory afferent neurons are critical components in the onset of prediabetes, promoting islet inflammation thorough altered glucose homeostasis and progressive β cell stress (5). Those involved in pancreas innervation have their cell bodies in the DRG end thoracic region. To find out if the functional defects described above could be translated into morphological changes during disease development, we first performed DRG histological studies of 32 weeks old non-diabetic NOD female mice. The results showed that some sensory afferent neurons of NOD mice display large single-membrane vacuoles in their cytoplasm (**Figure 1A**), which were not observed in 32 weeks old C57BL/6 mice (data not shown). Interestingly, in a surveyed sample from a NOD 26 weeks old female mouse, we found a DRG peripheral leukocyte infiltration (anecdotal observation detected in just one of the approximately 50 mice analyzed) (**Figure 1B**). To determine whether DRG cell vacuolization was the result of an autoimmune response, we performed histological studies in immunodeficient NOD.RAG-2^-/-^ mice. In these mice, vacuolization in DRG sensory neurons was also observed (**Figures 1C, D**), thus indicating that this injury was not the outcome of an autoimmune response but rather somewhat intrinsic to the NOD genetic background affecting sensory neurons. Moreover, our studies indicate that cytoplasmic vacuolization was already evident at two weeks of age in NOD.RAG-2^-/-^ mice (**Figure 1E**). Next, to know vacuolization onset in DRG sensory neurons and its potential connection with diabetes development, T7 to T11 DRGs from prediabetic six, nine and 12 weeks old and diabetic NOD mice were dissected. Cytoplasmic vacuoles in sensory neurons were observed at all ages without significant differences between age groups or diabetes occurrence (**Figure 1F**). The highest percentage of damage found was up to 3% of the total sensory neurons present in a DRG. Altogether, these results indicate that DRG sensory neuron damage already occurs at very early years of life and remains constant throughout life in NOD mice.

### DRG cells from NOD mice display gene transcriptional dysfunctions

The morphological alterations observed in NOD and NOD.Rag2^-/-^ mice could be affecting the physiological role of DRG neurons. The above results suggested that this suspected functional alteration of DRG sensory neurons was somewhat intrinsic to the NOD background. In order to determine the molecular elements involved in this disturbance, mRNA from DRG sensory neurons of three and 12 weeks old NOD, NOD.RAG-2^-/-^ and C57BL/6 mice was used for genome-wide profiling microarray analysis.

**Figure 2:**
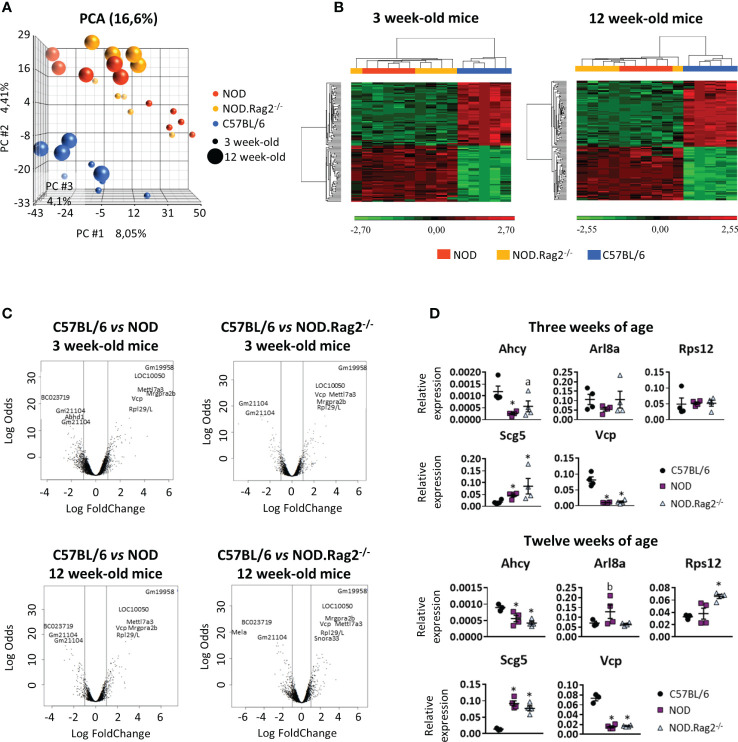
Comparison of the expression profiles of NOD, NOD-Rag2^-/-^ and C57BL/6 mice at 3 and 12 weeks of age. **(A)** PCA (Principal Component Analysis) representation of the expression profiles of the different strains; **(B)** Heatmaps showing a hierarchical clustering of DEGs in different mouse strains at 3 and 12 weeks of age. Clusters with low expression values are represented in green, while those with high expression are represented in red; **(C)** Volcano plots with significant genes graphically highlighted. These genes are arranged along dimensions of biological and statistical significance. The horizontal dimension is the fold change between the two groups (on a log scale, so that up- and downregulation appear symmetric), and the vertical axis represents the p–value from the moderated–test on a negative log scale, so smaller p–values appear higher up. The first axis indicates biological impact of the change. The second indicates the statistical evidence, or reliability of the change; **(D)** Comparative expression analysis of *Ahcy*, *Arl8a*, *Rps12*, *Scg5*, and *Vcp* genes in DRG cells from C57BL/6 *vs.* NOD or NOD.RAG2^-/-^ mice by RT-qPCR. In all experiments N=5. * symbol means: p<0.005.

Principal Component Analysis (PCA) was performed to determine the relationship between groups based on global gene expression patterns. Samples were clustered based on the variance of all gene expression measurements within the data set. Since several comparative analyses were performed according to the age and genetic background of the mice, common patterns of regulation between different experimental conditions were sought by using hierarchical gene clusters. In the transcriptomic analysis of the entire DRG genome by PCA analysis (**Figure 2A**), the age of mice separates in the first component indicating that some expression differences were age-related, whereas the mouse genetic background was resolved on the second component, in which C57BL/6 mice was clearly distinct from NOD and NOD.RAG-2^-/-^ mice. Thus, whereas NOD and NOD.RAG-2^-/-^ had a similar gene expression profile, C57BL/6 mice had a different gene expression profile either at three or 12 weeks of age (**Figure 2B**). Furthermore, no significant differences were observed in gene expression profiles at different ages within the same mouse strain.

### Gene expression of DRG cells is shaped by mouse genetic background but not age-related

For each comparison, a list of DEGs was obtained, considering as significant genes those with adjusted p-value<0.01 and |LogFC|>1 (**Supplementary Tables 1–3**). To find genes affected by two or more conditions, a multiple comparison analysis was performed (**Figure 2C**). The analysis showed that 80 and 86 genes were DEGs between C57BL/6, and NOD or NOD.RAG-2^-/-^ mice at three and 12 weeks of age, respectively (**Figures 2B, C** and **Table 1**).

**Table 1:** Biological process in which the DEG are involved, both at 3 and 12 weeks of age.

Biological process	3 weeks old mice			12 weeks old mice		
	Genes	Up or down	Proportion of DEGs	Genes	Up or down	Proportion of DEGs
**Innate immunity and inflammation**	*Fau* *Hist1h2b* *Ly86* *Mmp25* *Pianp*	Up Down Down Down Up	5/80	*Ahcy*, *Fau*, *Hist1h2b*, *Mmp25*	Up Up Down Down	4/86
**Adaptive immunity**	*Igk* *H2-T22* *Ifna4*	Down Down Down	3/80	*H2-T22*, *Ifna4*, *Nts*	Down Down Down	3/86
**Cell signalling**			8/80			8/86
**GCPR signalling**	*Mrgpra2a* *Mrgpra2b* *Mrgprb4* *Mrgprx1* *Olfr1269*	Down Down Down Down Down		*Mrgpra3/4*, *Myo7a*, *Nts*, *Mrgpra2a/2b*, *Olfr1269*, *Scg5*	Up Down Down Down Down Up	
**Neuropeptide signalling**	*Gabra2* *Scg5*	Up Up				
**Others**	*Srp54c*	Down		*Mid1*, *Srp54c*	Down Down	
**Transport**			7/80			3/86
**Axonal transport**	*Arl8a*	Down		*Arl8a*	Down	
**Ion transport and homeostasis**	*Slco1c1* *Trpc3* *Tmem203*	Up Down Down				
**Transmembrane transport**	*Slc35a5* *Slc39a8*	Down Up		*Slc39a8*	Up	
**Golgi to membrane transport**	*Tglon1*	Down				
**Retrograde transport**				*Heatr5b*	Down	
**Protein synthesis**	*Rpl29* *Rpl3* *Rpl34* *Rps12*	Down Up Down Down	4/80	*Rpl29*, *Rpl3*, *Rpl34*, *Rps12*	Down Up Down Down	4/86
**Protein maturation**	*Cst12* *Hddc3* *Mamdc2* *Mettl7a3*	Up Up Up Down	4/80	*Cst12*, *Mettl7a3*, *Tgm5*	Up Down Up	3/86
**Protein degradation (UBL pathway)**	*Cisd3* *Vcp*	Down Down	2/80	*Hspe1*, *Vcp*	Down Down	2/86
**Metabolism**	*Abhd1* *Ahcy* *Alad* *Gnpda1* *Gsto1* *Mmp25* *Ppp1r3e* *Scg5*	Up Up Up Up Up Down Down Up	8/80	*Abhd1*, *Ahcy*, *Fbp2*, *Gnpda1*, *Gsto1*, *Pla2g4e*, *Ppp1r3e*, *Scg5*	Up Up Up Up Up Up Down Up	8/86
**Cell cycle**	*Hjurp*, *Psmc3ip*, *Pttg1*	Down Up Down	3/80	*Hjurp*, *Psmc3ip*, *Pttg1*	Down Up Down	3/86
**DNA repair response**	*Pttg1*, *Vcp*	Down Down	2/80	*Pttg1*, *Vcp*	Down Down	2/86
**Transcription related proteins**	*Psmc3ip*, *Samd11*	Up Up	2/80	*Psmc3ip*, *Samd11*	Up Up	2/86
**Antioxidant and oxidative stress**	*Gsto1*	Up	1/80	*Gsto1*, *P4ha3*	Up Down	2/86
**Response to stimuli**			2/80			
**Insulin** **Osmotic stress**	*H2afz* *Map7*	Down Down				
**Cell differentiation and proliferation**	*Osr2* *Erdr1*	Up Up	2/80	*Map7*, *Lect1*	Down Down	2/86
**Cell death**	*Itgb3bp*, *Vcp*	Up Down	2/80	*Vcp*	Down	1/86
**Muscle proteins**	*Tpm3*, *Zfp125*	Up Down	2/80	*Tpm3*, *Zfp125*	Up Down	2/86
**Membrane proteins without a clear function defined**	*Ostc*, *Tmem254a*, *Tmem254b*, *Tmem254c*, *Tmem72*	Down Down Down Down Down	5/80	*Ostc*, *Nxpe2*, *Tmem132c*, *Tmem254a*, *Tmem254b*, *Tmem254c*, *Tmem40*	Down Down Down Down Down Down Up	7/86

Down: Gene expression in NOD or NOD.Rag2^-/-^ mice is lower than in C57BL/6 mice. Up: Gene expression in NOD or NOD.Rag2^-/-^ mice is higher than in C57BL/6 mice.

### Genes related to inflammation, and protein synthesis and folding are the most DEGs

Once DEGs were identified, we investigated their main biological category and putative involvement with T1D development. We carried out an enrichment analysis to identify if there was any altered biological category in mice with a predisposition to the development of T1D (**Table 1**).

Among the known 80 DEGs at 3 weeks of age, 30 identified sequences do not have their protein function described yet: one small nuclear RNA, two microRNAs, four validated and two predicted non-coding RNAs, six pseudogenes, and 16 predicted genes with no associated protein. Categorization into function based on information provided by literature, showed that the remaining 49 DEGs identified by microarray analysis correlated with inflammation (*Fau*, *Hist1h2b*, *Ly86, Mmp25* and *Pianp*) and adaptive immune response (*Igk*, *H2-T22* and *Ifna4*); signaling, mainly through G-Protein Coupled Receptors or GPCR (*Mrgpra2a, Mrgpra2b, Mrgprb4, Mrgprx1* and *Olfr1269*) but also other signaling pathways (*Gabra2* and *Scg5* -neuropeptide signaling- and *Srp54c*); transport (*Arl8a*- axonal transport -, *Slco1c1*, *Trpc3, Tmem203* –ion transport and homeostasis -, *Slc35a5*, *Slc39a8* – transmembrane transport- and *Tglon1* – Golgi to membrane transport); protein synthesis (*Rpl29*, *Rpl3*, *Rpl34* and *Rps12*), maturation (*Cisd3, Cst12*, *Hddc3*, *Mamdc2* and *Mettl7a3*) and degradation through the UBL pathway (*Vcp*); other metabolic processes (*Abhd1*, *Ahcy*, *Alad*, *Gnpda1*, *Gsto1*, *Mmp25, Ppp1r3e* and *Scg5*); cell cycle (*Hjurp*, *Psmc3ip* and *Pttg1*); DNA repair response (*Pttg1* and *Vcp*); transcription-related proteins (*Samd11*); antioxidant and oxidative stress proteins (*Gsto1*); cell differentiation and proliferation (*Osr2* and *Erdr1*) and cell death (*Itgb3bp* and *Vcp*). We also found two muscle proteins (*Tpm3* and *Zfp125*) and five membrane proteins without a clear function defined (*Ostc*, *Tmem254a*, *Tmem254b*, *Tmem254c* and *Tmem72*).

Similar results were observed at 12 weeks of age. Among the known 86 DEGs, 61 were also differentially expressed at three weeks of age, and 25 were differentially expressed only at this later pre-diabetic stage. We found nine pseudogenes, three non-coding RNA and two RIKEN cDNA, one small nucleolar RNA, 14 predicted genes and six sequences with no information. The remaining DEGs correlated with innate (*Ahcy*, *Fau*, *Hist1h2b* and *Mmp25*) and adaptive (*H2-T22*, *Ifna4* and *Nts*) immune responses; GPCR signaling (*Mrgpra3/4*, *Myo7a*, *Nts*, *Mrgpra2a/2b* and *Olfr1269*) but also other signaling pathways (*Scg5* -neuropeptide signaling pathway-, *Mid1* and *Srp54c*); transport (*Arl8a*- axonal transport -, *Heatr5b* –retrograde transport and endocytosis- and *Slc39a8* – transmembrane transport); protein synthesis (*Rpl29*, *Rpl3*, *Rpl34* and *Rps12*), maturation (*Cst12*, *Mettl7a3* and *Tgm5*) and degradation through the UBL pathway (*Hspe1* and *Vcp*); other metabolic processes (*Abhd1*, *Ahcy*, *Fbp2*, *Gnpda1*, *Gsto1*, *Pla2g4e*, *Ppp1r3e* and *Scg5*); cell cycle (*Hjurp*, *Psmc3ip* and *Pttg1*); DNA repair response (*Pttg1* and *Vcp*); transcription related proteins (*Psmc3ip* and *Samd11*); antioxidant and oxidative stress proteins (*Gsto1* and *P4ha3*); response to stimulus like insulin (*H2afz*) or osmotic stress (*Map7*); cell differentiation (*Map7* and *Lect1*) and cell death (*Vcp*). At twelve weeks of age, the muscle protein genes *Tpm3* and *Zfp125*, and the membrane protein genes *Ostc*, *Nxpe2*, *Tmem132c*, *Tmem254a*, *Tmem254b*, *Tmem254c* and *Tmem40* were also found to be differentially expressed.

Next, an Over Representation Analysis (ORA) and a Gene Set Enrichment Analysis (GSEA) were performed to detect any biological process, molecular function, cellular component, or pathway appearing more times than expected among these DEGs. By means of these analyses, a trend (FDR= 0.05-0.1) of gene transcript enrichment encoding ribosomal proteins at both 3 and 12 weeks of age was observed. At 12 weeks, a trend of transcript enrichment of genes related to antigen processing and presentation (FDR=0.1) was also found (**Table 2**).

**Table 2:** Enrichment analysis.

Enrich method	Type of enrichment	Enriched categories	Three weeks of age			Twelve weeks of age		
			Genes	p-value	FDR	Genes	p-value	FDR
ORA	Biological Process	Positive regulation of Tumor Necrosis Factor production	*Ccl19, H2-T23, Ccl2*	2.18e^-04^	0.85	*Ccl19, H2-T23*	6.78e^-03^	1
		Response to fatty acid	*Ccl19, Alad, Ccl2*	5.36e^-04^	0.85			
		Response to other organism	*Ccl19, Fau, H2-T23, Ifna4, Alad, Ccl2, Trim34a*	5.56e^-04^	0.85			
		Response to arsenic-containing substance	*Gsto1, Alad*	8.67e^-04^	0.85			
		Osteoblast proliferation	*Osr2, Npr3*	1.21e^-03^	0.85			
		Humoral Immune Response				*Fau, H2-T23, Ifna4*	2.06e^-03^	1
		Antigen processing and presentation				*Ccl19, H2-T22, H2-T23*	2.21e^-03^	1
		Peptide cross-linking				*Mamdc2, Tgm5*	6.34e^-03^	1
		Regulation of peptidase activity				*Mmp25*, ***Vcp*** *, Pttg1, Cst12*	9.95e^-03^	1
	Molecular function	MHC class I protein binding	*H2-T23*, ***Vcp***	3.91e^-04^	0.58	*H2-T23*, ***Vcp***	2.40e^-03^	0.43
		Peptidase regulator activity	*Mmp25*, ***Vcp*** *, Pttg1, Cst12*	1.36e^-03^	0.58	*Mmp25*, ***Vcp*** *, Pttg1, Cst12*	3.62e^-03^	0.43
		ADP binding				*Myo7a*, ***Vcp***	4.71e^-03^	0.43
		Antigen binding	*H2-T22, H2-T23*	1.37e^-03^	0.58	*H2-T22, H2-T23*	9.00e^-03^	0.61
		Unfolded Protein binding				*Hspe1*, ***Scg5***	0.03	1
		Lipase activity				*Pla2g4e, Abhd1*	0.04	1
		CCR chemokine receptor binding	*Ccl19, Ccl2*	1.71e^-03^	0.58			
		structural constituent of ribosome	*Fau, Rpl29, Rpl3*	3.41e^-03^	0.72	*Rpl29, Rpl3*	0.06	1
		Hydro-lyase activity	*Alad, L3hypdh*	4.82e^-03^	0.82			
	Cellular component	cytosolic ribosome	*Fau, Rpl29, Rpl3*	6.35e^-04^	0.64	*Fau, Rpl29, Rpl3*	5.76e^-04^	0.58
		endoplasmic reticulum	*H2-T23, Hsd17b7, Ccl2*, ***Vcp*** *, Ostc, Retsat, Mamdc2*	7.18e^-03^	0.99			
		Barr body	*H2afz*	8.03e^-03^	0.99	*H2afz*	7.77e^-03^	0.87
		Component of synaptic vesicle membrane	*Gabra2*	0.01	0.99			
		Cytosol				*Fau, Fbp2, Gsto1, Myo7a, Rpl29*, ***Vcp*** *, Rpl3, Pla2g4e*	5.01e^-03^	0.84
	Pathway_ Wikipathway	Cytoplasmic Ribosomal Proteins	*Fau, Rpl29*, ***Rps12*** *, Rpl3, Rpl32l, Rpl34*	3.86e^-06^	6.61e^-04^	*Fau, Rpl29*, ***Rps12*** *, Rpl3, Rpl32l, Rpl34*	2.92e^-07^	4.98e^-05^
	Pathway_ KEGG	Ribosome	*Fau, Rpl29*, ***Rps12*** *, Rpl3, Rpl34*	3.28e^-04^	9.82e^-02^	*Fau, Rpl29*, ***Rps12*** *, Rpl3, Rpl34*	1.87e^-04^	5.6e^-02^
	Pathway_ Reactome	rRNA processing	*Rpl29, Rpl3, Rpl34*	2.52e^-04^	0.1			
GSEA	Pathway_ Wikipathway	Cytoplasmic Ribosomal Proteins	*Rpl29*, ***Rps12*** *, Rpl34*	0.08	0.08	*Rpl29*, ***Rps12*** *, Rpl34*	0.17	0.17
	Pathway_ KEGG	Ribosome	*Rpl29*, ***Rps12*** *, Rpl34*	0.04	0.04	*Rpl29*, ***Rps12*** *, Rpl34*	0.05	0.05
	Pathway_ Reactome	Metabolism of proteins				*Rpl29*, ***Vcp*** *, Rpl34*	0.03	0.08

Downregulated genes are labelled in green. Upregulated genes are in red.

Next, to validate the expression profile, RT-qPCR analysis on DRG cells was performed in five DEGs between C57BL/6 and NOD or NOD.RAG2^-/-^ mice. Genes were selected according to low adj. p-value in both comparisons, association with an Idd locus and/or homology with the human gene higher than 80% (**Table 3**). Not all five genes showed the same gene expression pattern seen in the microarray analysis (**Figure 2D**). Consistent with the microarray assays, RT-qPCR analyses indicated that *Scg5* and *Vcp* genes were over and down-regulated, respectively, both at three and 12 weeks of age in NOD and NOD.RAG-2^-/-^ mice when compared to C57BL/6 mice. No significant differences between groups for *Arl8a* and *Rps12* genes expression were observed by RT-qPCR analysis, although for *Arl8a* gene a trend toward a statistical significance consistent with microarray assays at three weeks of age was observed when comparing NOD and C57BL/6 mice. Lastly, the RT-qPCR results showed the opposite expression pattern to the microarray results for the *Ahcy* gene.

**Table 3:** Main characteristics of genes selected for RT-qPCR analysis.

Selected genes		NOD and NOD.Rag2^-/-^ expression levels compared to C57BL/6 expression levels	Biological process	Idd locus	Human and mice genes homology (%)
Symbol	Name				
*Ahcy*	*S-adenosyl-homocysteine hydrolase*	↑	Homocysteine biosynthesis	Idd13	91
*Arl8a*	*ADP-ribosylation factor-like 8A*	↓	Chromosome segregation (cell cycle)	Idd5.4	93
*Rps12*	*Ribosomal protein S12*	↓	Protein synthesis	Idd13	90
*Scg5*	*Secretogranin V*	↑	Metabolism	Idd13 (±0.5Mb)	81
*Vcp*	*Valosin Containing Protein*	↓	UBL Pathway	–	89

↑, Upregulated gene expression in NOD or NOD.Rag2-/- compared to C57BL/6 mice. ↓, Downregulated gene expression in NOD or NOD.Rag2-/- compared to C57BL/6 mice.

### Peripheral blood leukocytes from NOD mice also show gene expression defects

**Figure 3:**
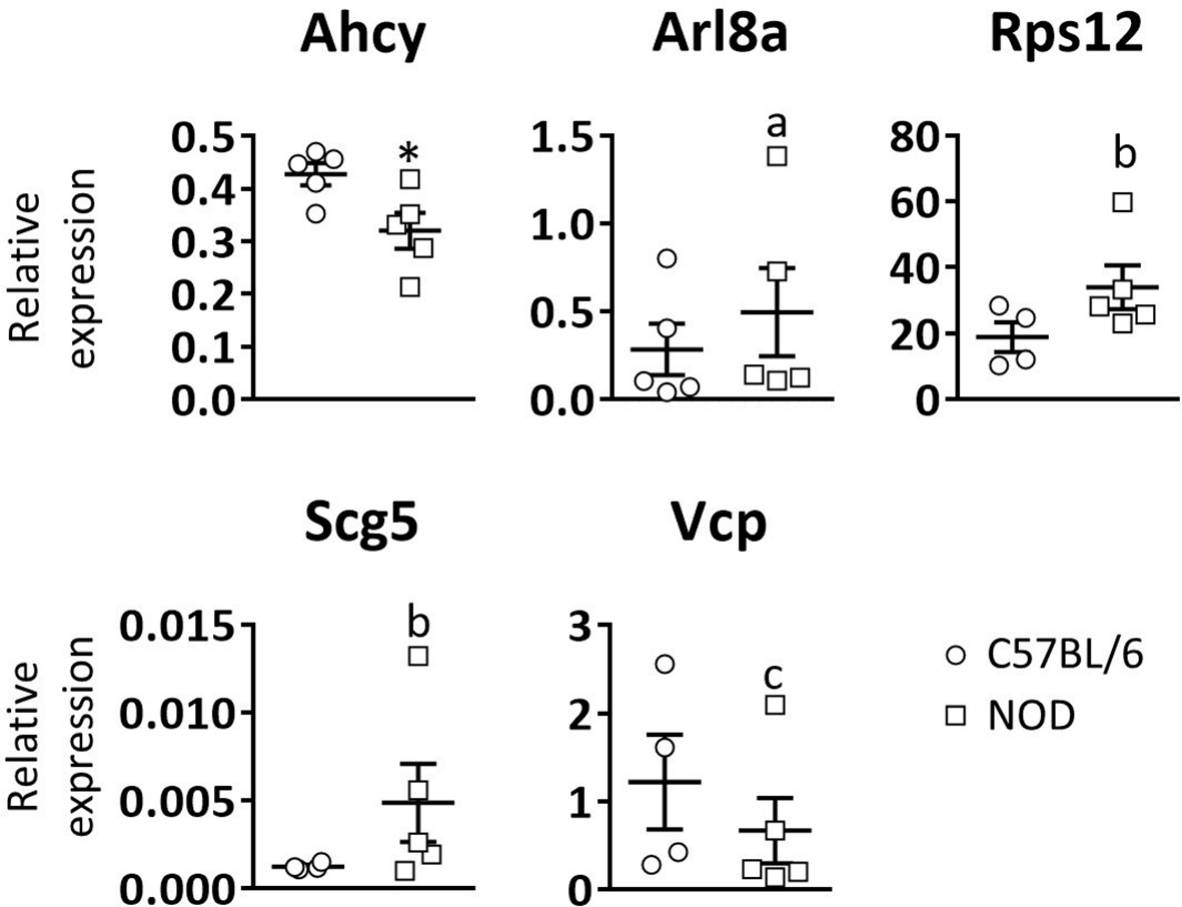
Relative expression levels of selected genes in blood leukocytes. Comparative expression analysis of *Ahcy*, *Arl8a*, *Rps12*, *Scg5*, and *Vcp* genes in white blood cells from 3 weeks old C57BL/6 and NOD mice by RT-qPCR. N=5. * symbol means: p<0.005. Letter “a” means p=0.15; letter “b” means p=0.09; letter “c” means p=0.14.

Next, to see if these gene expression defects are unique to DRG and pancreatic β-cells, or a common trait found in other NOD mouse cell lineages, RT-qPCR analysis of the five selected genes was performed in white blood cells of three weeks old C57BL/6 and NOD female mice (**Figure 3**). Only significant differences were observed for the *Ahcy* gene. A trend towards significance in the expression of the *Rps12*, *Scg5*, and *Vcp* genes was found, which is consistent with the results observed in DRG cells.

## Discussion

Although pancreatic β-cells are the main target of the autoimmune attack in T1D (26, 27), the presence of autoreactive T and B lymphocytes against the nervous system has been previously described (4, 28, 29). This response against neuronal autoantigens has been reported in early stages of diabetes, suggesting that it may precede the autoimmune attack against pancreatic β-cells (5).

Here, we show that some DRG sensory neurons have vacuoles in their cytoplasm. This alteration remains constant at different ages, being already detected at two weeks of age, thus indicating that it is something intrinsic to the NOD strain. As we observed this cytoplasm vacuolization of DRG cells in NOD.RAG-2^-/-^ mice, we can conclude that it is not a consequence of an autoreactive immune response (30).

Several studies have linked the accumulation of lysosomes and autophagic vacuoles in the cytoplasm with the onset of different neurodegenerative diseases, such as Alzheimer’s (31, 32), Parkinson’s (33), Huntington’s (34), and Amyotrophic Lateral Sclerosis (35), among others (36). The significance of the presence of vacuoles in DRGs cells is a controversial issue. Some authors consider that they have pathological relevance while others suggest they could be considered artifacts or spontaneous changes (37). In our studies, electron microscopy analyses indicated that the vacuoles have a single membrane, thus ruling out the hypothesis of an autophagic process, in which there should be double membrane vesicles. Such alterations may be due to excitotoxicity (38), which is a characteristic phenomenon of neurodegenerative processes. A similar alteration has been reported in the DRG cells of diabetic BB rats, another model of spontaneous T1D. It has been postulated that it may be caused by a deterioration in the cytoskeleton protein synthesis (39). However, specific assays such as neurite outgrowth should be performed to confirm that this vacuolization is related to a neurodegenerative process. Vacuole formation in DRGs can also occur in the context of metabolic dysfunction. Under cellular stress conditions, such as that found in the diabetogenic process, DRG cells may experience changes in their metabolism and in the way they handle nutrients and waste (24). Metabolic dysfunction in DRGs can lead to abnormal accumulation of lipids and other waste products within cells, which can result in vacuole formation. These vacuoles can disrupt the normal structure and function of DRG neurons, which may have consequences for sensory signal transmission and overall nerve function. Furthermore, the formation of vacuoles in the DRGs has been linked to the development of diabetic neuropathy, which is thought to be caused by a combination of metabolic dysfunction, inflammation, and oxidative stress (23). Our results suggest that this formation of vacuoles in DRG cells can occur early in life and previous to the onset of diabetic neuropathy, being the result of intrinsic functional alterations in these sensory neurons. These disturbances would lead to the accumulation of vacuoles in the DRG sensory neurons, initiating a neurodegenerative process that, ultimately, could lead to the appearance of diabetic neuropathy.

In the present study, we performed mRNA expression analysis to determine the putative cause and/or the molecules involved in this suspected neurodegenerative process. The results showed that DRG cells from NOD (and NOD.RAG-2^-/-^) mice have a different gene expression pattern compared to C57BL/6 mice, indicating that DRG cells from NOD mice have a functional impairment. Microarray analyses indicate that these functional changes mainly affect pathways of inflammatory processes and immune response, as well as protein synthesis and folding. However, the involvement of other functional pathways is also evident. To validate the results obtained by the microarray analysis, a RT-qPCR assay of five DEGs was performed. These genes were selected according to low adj. p-value, association with an Idd locus and/or homology with the human genes. As described in the *Results* section, RT-qPCR analyses partially support the microarray results. Only two of the five selected genes behave as expected, both at three and 12 weeks. The other genes show no significant expression differences between mice groups by RT-qPCR and *Ahcy* gene despite showing an opposite behavior in the two assays. Besides that, this study has allowed us to find that *Scg5* and *Vcp* gene transcription is up and down-expressed in DRG cells from NOD and NOD.RAG-2^-/-^ mice respectively compared to C57BL/6 mice. This has been confirmed by two different assays indicating that they are good candidate genes for further investigation in T1D. Although the *Ahcy* gene does not behave as expected in RT-qPCR results, it shows different expression levels between mice groups. Either raising or lowering the transcription, *Ahcy* gene expression is altered, and it may also be a good candidate gene for further investigation.

Taken together, our results support the hypothesis that there is a relation between neuronal degeneration and AD development. Some studies describe a dysfunction in TRPV1^+^ sensory neurons in NOD mice, which inhibits their activation and the neuropeptide release to the pancreatic islet cells (15). Moreover, *Trpv1* gene is located on an AD susceptibility locus in NOD mice (Idd4.1). TRPV1^+^ mutations have also been related to T1D susceptibility in humans (14). *Arl8a* is a small GTPase involved in equal segregation of chromosomes. Reducing its activity by either overexpression of dominant-negative mutants or RNAi induces abnormal morphology in the chromosome segregation (40). It has been recently demonstrated by mass spectrometry analysis that it interacts with TRPV1 receptors (41), which suggests an unknown role for this protein. Although our results are not clear, *Arl8a* gene seems to be down-expressed in DRG cells by microarray analysis, supporting its new role in TRPV1 signaling in NOD mice DRG cells. *Scg5* is the chaperone of pro-Protein Convertase 2 (pro-PC2). PC2, together with Protein Convertase 1 (PC1) and Protein Convertase 3 (PC3), is involved in pro-hormones and pro-neuropeptides processing. Scg5 also facilitates PC2 transport from the ER to later compartments of the secretory pathway (42–44). Scg5 is over-expressed in NOD mice DRG cells, suggesting an altered neuropeptide processing and signaling. Scg5 is also over-expressed in NOD mice white blood cells, which suggests a new unknown role in the characteristic autoimmune response of T1D. However, more studies are needed to completely understand the involvement of this neurodegeneration in T1D development. There is a complex association between the nervous system and the immune system through the action of neurotransmitters. In addition, the neuronal alterations may expose autoantigens that, in physiological conditions, would not be visible to the immune system. This would favor an autoimmune response in those individuals susceptible to develop T1D. This hypothesis is based on the autoimmune response against neuroendocrine antigens observed in T1D, which are targets of the immune system at early stages of T1D (4, 28). A clear example is the presence of autoreactive lymphocytes against Glial Fibrillary Acidic Protein (GFAP) in mice before three weeks of age (45).

Although β cells undergo high ER stress levels in normal conditions, it has been postulated that a disruption of ER homeostasis may contribute to β cell dysfunction and diabetes. In fact, some studies have demonstrated that mutations in genes critical for ER function result in β cell failure and early onset of T1D (31, 46). Our microarray results suggest that DRG cells of NOD and NOD.RAG-2^-/-^ mice undergo ER stress, which evoke that ER stress is not only a problem existing in β cells but also in the PNS cells, the other known target of T1D. The origin of this stress is unknown, but it may be related to an alteration of the calcium homeostasis which would cause a dysfunction of the ER chaperones and, consequently, the accumulation of unfolded and misfolded proteins. DRG cells of NOD and NOD.RAG-2^-/-^ mice present alterations in the UBL pathway and, as a result, these proteins cannot be degraded and keep accumulating. This process may facilitate the autoantigen presentation in a context of stress and inflammation. This hypothesis is strengthened by the RT-qPCR results, where we found low expression of *Rps12* and *Vcp* both in DRG and white blood cells. *Rps12* plays important regulatory functions in the ribosome and helps to maintain a normal protein synthetic rate. *Vcp* is a multifunction protein. It is involved in the formation of the transitional endoplasmic reticulum (tER) and in ubiquitinated proteins degradation (UBL pathway). It is also involved in DNA damage response, being recruited to double strand break sites (DSBs) in a RNF8 (Ring Finger protein 8) and RNF168 (Ring Finger protein 168) dependent manner and promoting the recruitment of TP53BP1 (Tumor Protein p53 Binding Protein 1) at DNA damage sites. In association with NPLOC4 (Nuclear protein localization protein 4 homolog) and UFD1L (Ubiquitin fusion degradation protein 1 homolog), it regulates spindle disassembly at the end of mitosis and is necessary for the formation of a closed nuclear envelope. In terms of immune response, it has been described that *Vcp* is a potential substrate of Protein Tyrosine Phosphatase Non-receptor type 22 (PTPN22), a protein-tyrosine phosphatase involved in TCR-signaling pathway. Mutations of PTPN22 have been associated with a range of autoimmune diseases including T1D (47). Moreover, *Vcp* interacts selectively and non-covalently with major histocompatibility complex class I molecules, which are responsible for antigen presentation (48).

Finally, as regards pancreatic islets innervation, dysfunction of the PNS may impede the ability of the PNS to regulate beta cell function, impairing insulin secretion from these cells. This could partly explain why insulin synthesis is compromised in patients with T1D despite maintaining nearly 50% of the original beta cell mass (49).

In conclusion, our results show an impaired functionality of DRG cells in NOD mice. This disturbance affects the expression of genes mainly related to inflammation and the immune response, and to the synthesis and folding of proteins, among others. These results suggest that DRG cells undergo ER stress and the consequent triggering of the UPR. However, UPR could fail to solve this ER stress, resulting in the accumulation of misfolded proteins that may be observed in some DRGs with the presence of vacuoles. These vacuoles can disrupt the normal structure and function of DRG neurons, which may have consequences for sensory signal transmission and overall nerve function. This PNS dysfunction may predispose it to be targeted by the immune system, leading to T1D. If our hypothesis is correct, DRG cells of T1D susceptible individuals would also present alterations in the transcription in a wide range of genes. Moreover, our results suggest that functional genetic defects could be general and affect multiple body cell lines. One candidate gene is *Vcp* which, according to our results in NOD mice, is also low transcribed in mononuclear blood cells. Since it is not possible to access the DRG cells *in vivo*, *Vcp* gene expression analysis in the white blood cell population could be a simple and easy way to screen for T1D susceptibility individuals prior to disease onset. Future studies in this direction using patient samples will be crucial to confirm or reject this hypothesis.

## Data availability statement

The original contributions presented in the study are publicly available. This data can be found here: https://figshare.com/articles/figure/Histological_analysis_of_DRG_cells_from_NOD_mice/22820789.

## Ethics statement

This study was carried out in accordance with the principles of the Basel Declaration and recommendations of the Catalan Government (Generalitat de Catalunya) concerning the protection of animals for experimentation. The protocol was approved by the Committee on the Ethics of Research in Animal Experimentation of the University of Lleida, Spain. Protocol #: CEEA 02-04/16.

## Author contributions

MC-P, BA, LE-M, ER-M, AS and AC, researched data. MC-P and JV wrote the manuscript and edited the manuscript. CM, TS, DM, MV-P, AS, AC and JE contributed to discussion. JV is the guarantor of this work and, as such, takes responsibility for the integrity of the data and the accuracy of the data analysis. All authors contributed to the article and approved the submitted version.
