# Supplementary material for: NOD mouse dorsal root ganglia display morphological and gene expression defects before and during autoimmune diabetes development
Source: Front Endocrinol (Lausanne). 2023 Jun 2;14:1176566. doi: 10.3389/fendo.2023.1176566 (PMC10272810; doi:10.3389/fendo.2023.1176566)
Supplement: Supplementary file 1 [file Table_1.docx]

**Supplementary Table I. DEGs in each comparison, at 3 and 12 weeks of age.**

| **Genes** | | **C57BL/6 vs. NOD**  **3 weeks of age** | | | **C57BL/6 vs. NOD.RAG2^-/-^**  **3 weeks of age** | | | **C57BL/6 vs. NOD**  **12 weeks of age** | | | **C57BL/6 vs. NOD.RAG2^-/-^**  **12 weeks of age** | | |
| --- | --- | --- | --- | --- | --- | --- | --- | --- | --- | --- | --- | --- | --- |
| **Symbol** | **Name** | **adjusted p-value** | **logFC** | **Up or Down** | **adjusted p-value** | **logFC** | **Up or Down** | **adjusted p-value** | **logFC** | **Up or Down** | **adjusted p-value** | **logFC** | **Up or Down** |
| *Abhd1* | *Abhydrolase domain containing 1* | 4,93E-08 | -2,08 | Up | 8,27E-07 | -1,75 | Up | 1,28E-05 | -1,48 | Up | 6,35E-06 | -1,55 | Up |
| *Ahcy* | *S-adenosyl-homocysteine hydrolase* | 6,31E-04 | -2,03 | Up | 0,01 | -1,55 | Up | 6,20E-03 | -1,67 | Up | 5,71E-03 | -1,68 | Up |
| *Alad* | *Aminolevulinate, delta-, dehydratase* | 7,73E-04 | -1,31 | Up | 2,08E-03 | -1,23 | Up | 0,38 | -0,52 |  | 7,30E-03 | -1,07 | Up |
| *Arl8a* | *ADP-ribosylation factor-like 8A* | 1,16E-04 | 1,49 | Down | 3,87E-04 | 1,38 | Down | 2,66E-04 | 1,4 | Down | 1,96E-05 | 1,7 | Down |
| *Arntl2* | *Aryl hydrocarbon receptor nuclear translocator-like 2* | 0,036 | 0,6 |  | 0,24 | 0,47 |  | 9,53E-03 | 0,84 | Down* | 1,34E-03 | 1,02 | Down |
| *Ccl19* | *Chemokine (C-C motif) ligand 19* | 2,94E-09 | -2,6 | Up | 6,59E-11 | -3,09 | Up | 1,80E-10 | -2,96 | Up | 1,12E-09 | -2,7 | Up |
| *Cisd3* | *CDGSH iron sulfur domain 3* | 1,69E-04 | 1,24 | Down | 1,64E-03 | 1,04 | Down | 0,02 | 0,81 | Down* | 1,82E-04 | 1,23 | Down |
| *Cst12* | *Cystatin 12* | 7,85E-04 | -1,26 | Up | 2,88E-03 | -1,15 | Up | 1,15E-04 | -1,48 | Up | 3,15E-04 | -1,38 | Up |
| *Fau* | *Finkel-Biskis-Reilly murine sarcoma virus (FBR-MuSV) ubiquitously expressed (fox derived)* | 1,77E-04 | -1,22 | Up | 1,53E-03 | -1,05 | Up | 1,24E-05 | -1,49 | Up | 2,83E-05 | -1,4 | Up |
| *Fbp2* | *Fructose biphosphatase 2* | 0,043 | -0,76 |  | 0,01 | -1,11 | Up | 2,04E-04 | -1,57 | Up | 5,29E-04 | -1,47 | Up |
| *Gabra2* | *Gamma-aminobutyric acid (GABA) A receptor, subunit alpha 2* | 4,98E-03 | -1,19 | Up | 4,90E-04 | -1,54 | Up | 1,52E-03 | -1,4 | Up | 0,08 | -0,822 | Up* |
| *Gnpda1* | *Glucosamine-6-phosphate isomerase 1* | 9,59E-06 | -2,24 | Up | 6,37E-05 | -1,97 | Up | 2,94E-06 | -2,39 | Up | 6,41E-07 | -2,61 | Up |
| *Gsto1* | *glutathione S-transferase omega 1* | 8,86E-05 | -1,32 | Up | 5,49E-04 | -1,16 | Up | 6,80E-04 | -1,14 | Up | 8,93E-04 | -1,11 | Up |
| *H2afz* | *H2A histone family, member Z* | 0,02 | 1,05 | Down* | 0,04 | 1,11 | Down* | 1,42E-03 | 1,61 | Down | 5,71E-03 | 1,41 | Down |
| *H2-T22* | *Histocompatibility 2, T region locus 22* | 1,52E-06 | 2,87 | Down | 3,99E-07 | 3,09 | Down | 4,45E-07 | 3,08 | Down | 6,35E-06 | 2,63 | Down |
| *Hddc3* | *HD domain containing 3* | 6,35E-04 | -1,48 | Up | 1,55E-03 | -1,41 | Up | 0,02 | -1,06 | Up* | 0,02 | -1,05 | Up* |
| *Heatr5b* | *HEAT repeat containing 5B* | 0,01 | 0,87 | Down* | 4,75E-04 | 1,30 | Down | 1,73E-04 | 1,41 | Down | 2,41E-04 | 1,37 | Down |
| *Hist1h2b* | *H2B clustered histone 4* | 2,51E-05 | 1,78 | Down | 2,52E-06 | 2,06 | Down | 6,68E-05 | 1,67 | Down | 8,13E-07 | 2,18 | Down |
| *Hjurp* | *Holliday junction recognition protein* | 3,58E-03 | 1,06 | Down | 2,46E-03 | 1,14 | Down | 2,25E-04 | 1,37 | Down | 1,71E-03 | 1,17 | Down |
| *Hspe1* | *Heat shock protein l (chaperonin 10)* | 0,01 | 1,03 | Down | 1,73E-03 | 1,34 | Down | 1,61E-04 | 1,6 | Down | 9,85E-04 | 1,4 | Down |
| *Ifna4* | *Interferon alpha 4* | 5,64E-05 | 1,83 | Down | 1,40E-04 | 1,73 | Down | 1,58E-03 | 1,42 | Down | 4,06E-05 | 1,88 | Down |
| *Itgb3bp* | *Integrin beta 3 binding protein (beta3-endonexin)* | 2,42E-03 | -1,09 | Up | 8,28E-03 | -1,00 | Up | 0,21 | -0,59 |  | 0,11 | -0,63 |  |
| *Lect1* | *Leucocyte cell derived chemotaxin 1* | 0,02 | 0,98 | Down* | 0,01 | 1,18 | Down | 7,12E-04 | 1,51 | Down | 9,16E-04 | 1,48 | Down |
| *Ly86* | *Lymphocyte antigen 86* | 6,89E-03 | 1,00 | Down | 2,02E-03 | 1,19 | Down | 4,92E-03 | 1,09 | Down | 0,02 | 0,89 | Down* |
| *Mamdc2* | *MAM domain containing 2* | 3,50E-05 | -1,79 | Up | 1,93E-03 | -1,33 | Up | 2,64E-03 | -1,28 | Up | 0,02 | -1,05 | Up* |
| *Map7* | *Microtubule-associated protein 7* | 0,02 | 1,02 | Down | 0,01 | 1,21 | Down | 7,34E-03 | 1,21 | Down | 0,01 | 1,25 | Down |
| *Mettl7a3* | *methyltransferase like 7A3* | 3,63E-12 | 4,6 | Down | 2,90E-12 | 4,59 | Down | 3,41E-12 | 4,61 | Down | 4,47E-12 | 4,5 | Down |
| *Mid1* | *Midline 1* | 4,20E-03 | 1,07 | Down | 0,32 | 0,5 |  | 8,53E-04 | 1,27 | Down | 1,61E-03 | 1,21 | Down |
| *Mmp25* | *Matrix metallopeptidase 25* | 2,12E-04 | 1,36 | Down | 6,76E-03 | 1,02 | Down | 1,92E-04 | 1,37 | Down | 2,04E-04 | 1,37 | Down |
| *Mrgpra2a/b* | *MAS-related GPR, member A2B* | 9,87E-07 | 1,64 | Down | 4,01E-11 | 3,93 | Down | 1,56E-11 | 4,13 | Down | 4,47E-12 | 4,4 | Down |
| *Mrgpra3-4* | *MAS-related GPR, member A3/A4* | 0,2 | -0,4 |  | 0,06 | -0,74 |  | 5,71E-05 | -1,47 | Up | 1,36E-03 | -1,16 | Up |
| *Mrgprb4* | *MAS-related GPR, member B4* | 2,13E-05 | 2,03 | Down | 5,49E-05 | 1,91 | Down | 0,16 | 0,78 |  | 0,15 | 0,69 |  |
| *Mrgprx1* | *MAS-related GPR, member X1* | 3,25E-06 | 1,79 | Down | 4,64E-04 | 1,28 | Down | 0,29 | 0,53 |  | 0,06 | 0,72 |  |
| *Myo7a* | *Myosin VIIA* | 0,06 | 0,58 |  | 0,35 | 0,44 |  | 7,47E-04 | 1,18 | Down | 7,40E-04 | 1,19 | Down |
| *Nts* | *Neurotensin* | 0,33 | 0,43 |  | 0,1 | 0,98 |  | 1,39E-03 | 1,67 | Down | 6,32E-03 | 1,43 | Down |
| *Nxpe2* | *Neurexophilin and PC-esterase domain family, member 2* | 0,02 | 0,89 | Down* | 0,01 | 1,08 | Down | 6,64E-03 | 1,08 | Down | 2,69E-03 | 1,18 | Down |
| *Olfr1269* | *Olfactory receptor 1269* | 1,91E-06 | 1,85 | Down | 1,88E-07 | 2,11 | Down | 2,04E-04 | 1,35 | Down | 1,79E-03 | 1,14 | Down |
| *Osr2* | *Odd-skipped related 2* | 3,52E-04 | -1,66 | Up | 1,01E-03 | -1,57 | Up | 0,99 | -0,002 |  | 0,69 | -0,31 |  |
| *Ostc* | *Oligosaccharyl transferase complex subunit* | 4,72E-05 | 1,54 | Down | 6,02R-06 | 1,77 | Down | 1,06E-04 | 1,46 | Down | 4,27E-05 | 1,55 | Down |
| *P4ha3* | *Procollagen-proline, 2-oxoglutarate 4-dioxygenase (proline 4-hydroxylase), alpha polypeptide III* | 0,052 | 0,65 |  | 0,26 | 0,54 |  | 6,62E-03 | 1,05 | Down | 7,18E-03 | 1,03 | Down |
| *Pianp* | *PILR alpha associated neural protein* | 2,08E-03 | -1,09 | Up | 2,86E-03 | -1,10 | Up | 2,86E-03 | -1,10 | Up | 0,03 | -0,83 | Down* |
| *Pisd-ps3* | *Phosphatidylserine decarboxylase, pseudogene 3* | 3,85E-05 | 2,92 | Down | 3,83E-03 | 2,05 | Down | 8,93E-05 | 2,75 | Down | 2,21E-05 | 3,04 | Down |
| *Pla2g4e* | *phospholipase A2, groDown IVE* | 0,013 | -0,73 |  | 1,57E-03 | -1,00 | Up | 7,75E-05 | -1,25 | Up | 2,86E-04 | -1,14 | Up |
| *Ppp1r3e* | *Protein phosphatase 1, regulatory (inhibitor) subunit 3E* | 2,26E-04 | 1,31 | Down | 2,34E-04 | 1,33 | Down | 1,06E-05 | 1,65 | Down | 3,34E-06 | 1,77 | Down |
| *Psmc3ip* | *Proteasome (prosome, macropain) 26S subunit, ATPase 3, interacting protein* | 1,50E-03 | -1,29 | Up | 4,75E-03 | -1,19 | Up | 8,35E-03 | -1,12 | Up | 1,48E-03 | -1,32 | Up |
| *Pttg1* | *Pituitary tumor-transforming gene 1* | 6,90E-07 | 2,11 | Down | 2,48E-06 | 1,96 | Down | 4,02E-09 | 2,74 | Down | 4,35E-09 | 2,74 | Down |
| *Rian* | *RNA imprinted and accumulated in nucleus* | 6,49E-05 | 2,43 | Down | 1,06E-04 | 2,38 | Down | 3,26E-04 | 2,16 | Down | 1,47E-05 | 2,71 | Down |
| *Rpl29* | *Ribosomal Protein L29* | 2,02E-09 | 3,35 | Down | 2,58E-10 | 3,65 | Down | 2,57E-10 | 3,65 | Down | 2,72E-10 | 3,64 | Down |
| *Rpl3* | *Ribosomal protein L3* | 1,72E-03 | -1,38 | Up | 4,95E-05 | -1,88 | Up | 2,90E-05 | -1,92 | Up | 1,14E-03 | -1,46 | Up |
| *Rpl32* | *Ribosomal protein L32* | 0,033 | -0,81 | Up* | 0,01 | -1,06 | Up | 4,83E-06 | -2,85 | Up | 1,43E-04 | -2,26 | Up |
| *Rpl34* | *Ribosomal protein L34* | 1,80E-04 | 2,15 | Down | 1,86E-05 | 2,57 | Down | 4,33E-05 | 2,4 | Down | 3,98E-03 | 1,67 | Down |
| *Rps12* | *Ribosomal protein S12* | 7,31E-08 | 2,75 | Down | 1,11E-08 | 2,98 | Down | 1,72E-09 | 3,26 | Down | 1,60E-09 | 3,29 | Down |
| *Rps19-ps4* | *Ribosomal protein S19, pseudogene 4* | 6,77E-04 | -1,15 | Up | 0,02 | -0,83 | Up* | 2,65E-03 | -1,04 | Up | 8,33E-05 | -1,37 | Up |
| *Samd11* | *Sterile alpha motif domain containing 11* | 1,93E-04 | -1,44 | Up | 3,91E-05 | -1,65 | Up | 7,43E-07 | -2,08 | Up | 6,35E-06 | -1,84 | Up |
| *Scg5* | *Secretogranin V* | 1,16E-04 | -1,20 | Up | 7,70E-06 | -1,46 | Up | 1,70E-05 | -1,38 | Up | 2,38E-05 | -1,35 | Up |
| *Slc35a5* | *Solute carrier family 35, member A5* | 1,95E-03 | 1,08 | Down | 1,20E-03 | 1,17 | Down | 1,07E-03 | 1,17 | Down | 5,55E-03 | 1,00 | Down |
| *Slc39a8* | *Solute carrier family 39 (metal ion transporter), member 8* | 2,93E-04 | -1,26 | Up | 3,87E-04 | -1,25 | Up | 4,75E-05 | -1,45 | Up | 7,51E-06 | -1,64 | Up |
| *Slco1c1* | *Solute carrier organic anion transporter family, member 1c1* | 1,95E-03 | -1,53 | Up | 5,20E-04 | -1,77 | Up | 0,01 | -1,32 | Up | 0,03 | -1,12 | Up* |
| *Srp54c* | *Signal recognition particle 54C* | 2,21E-03 | 0,92 | Down* | 2,71E-04 | 1,12 | Down | 5,89E-05 | 1,27 | Down | 3,32E-04 | 1,11 | Down |
| *Tdg* | *Thymine DNA* | 1,41E-05 | -2,28 | Up | 2,13E-03 | -1,56 | Up | 1,08E-03 | -1,64 | Up | 3,12E-03 | -1,49 | Up |
| *Tgm5* | *Transglutaminase 5* | 0,07 | -0,64 |  | 0,02 | -1,02 | Up* | 8,01E-05 | -1,61 | Up | 1,48E-03 | -1,28 | Up |
| *Tgoln1-2* | *Trans-golgi network protein* | 2,12E-04 | 1,07 | Down | 1,62E-03 | 0,93 | Down* | 0,02 | 0,7 |  | 3,98E-03 | 0,84 | Down* |
| *Tmem132c* | *Transmembrane protein 132C* | 8,72E-04 | 1,16 | Down | 0,01 | 0,88 | Down* | 4,61E-03 | 1,02 | Down | 1,49E-03 | 1,13 | Down |
| *Tmem203* | *Transmembrane protein 203* | 1,72E-03 | -0,85 | Down* | 6,56E-03 | -0,77 |  | 0,0005 | -0,96 | Down* | 0,001 | -0,86 | Down* |
| *Tmem254a/b/c* | *Transmembrane protein 254* | 3,36E-05 | 1,34 | Down | 1,25E-05 | 1,39 | Down | 7,29E-05 | 1,28 | Down | 4,41E-05 | 1,27 | Down |
| *Tmem40* | *Transmembrane protein 40* | 0,03 | -0,81 | Up* | 2,07E-04 | -1,55 | Up | 2,07E-04 | -1,55 | Up | 2,80E-03 | -1,25 | Up |
| *Tmem72* | *Transmembrane protein 72* | 6,25E-04 | 1,20 | Down | 2,88E-03 | 1,07 | Down | 2,88E-03 | 1,07 | Down | 8,93E-04 | 1,18 | Down |
| *Tpm3* | *Tropomyosin 3, gamma* | 4,86E-07 | -2,12 | Up | 7,81E-05 | -1,53 | Up | 9,47E-06 | -1,78 | Up | 1,96E-05 | -1,69 | Up |
| *Trpc3* | *Transient receptor potential cation channel, subfamily C, member 3* | 7,27E-04 | 1,11 | Down | 3,27E-03 | 1 | Down | 0,1874 | 0,55 |  | 0,03 | 0,76 |  |
| *Vcp* | *Valosin containing protein* | 2,77E-11 | 2,99 | Down | 2,90E-12 | 3,34 | Down | 1,92E-11 | 3,04 | Down | 8,00E-12 | 3,15 | Down |
| *Zfp125* | *Zinc finger protein 125* | 1,76E-04 | 2,78 | Down | 1,32E-03 | 2,39 | Down | 1,54E-04 | 2,84 | Down | 7,40E-04 | 2,51 | Down |

Differences were considered significant when the adjusted p-value was less than 0.01 and the |LogFC| was greater than 1. Down: Expression in NOD or NOD.Rag2^-/-^ mice is lower than in C57BL/6 mice. Up: Expression in NOD or NOD.Rag2^-/-^ mice is higher than in C57BL/6 mice. Non-significant values are in red. The *asterisk* marks those values with a tendency to significance.

**Supplementary Table II. DEGs in each comparison, at 3 and 12 weeks of age.**

| **Genes** | | **NOD vs. NOD.RAG2^-/-^**  **3 weeks of age** | | | **NOD vs. NOD.RAG2^-/-^**  **12 weeks of age** | | |
| --- | --- | --- | --- | --- | --- | --- | --- |
| **Symbol** | **Name** | **adjusted p-value** | **logFC** | **Up or Down** | **adjusted p-value** | **logFC** | **Up or Down** |
| *Ccl8* | *Chemokine (C-C motif) ligand 8* | 7,65E-03 | 1,67 | Down | 0,45 | -0,88 |  |
| *Erdr1* | *Erythroid differentiation regulator 1 x* | 8,64E-03 | -1,78 | Up | 0,85 | 0,16 |  |
| *Igk* | *Immunoglobulin kappa chain complex* | 7,65E-03 | 4,18 | Down | 0,03 | 3,32 | Down* |
| *Mela* | *Melanoma antigen* | 1,18E-03 | -3,59 | Up | 5,19E-09 | -6,56 | Up |
| *Olfr1034* | *Olfactory receptor family 5 subfamily M member 9* | 7,38E-03 | -3,55 | Up | 9,12E-03 | 1,27 | Down |
| *Slc37a1* | *Solutee carrier family 37 (glycerol-3-phosphate transporter), member 1* | 0,18 | 0,68 |  | 9,12E-03 | -3,41 | Up |

Differences were considered significant when the adjusted p-value was less than 0.01 and the |LogFC| was greater than 1. Down: Expression in NOD.Rag2^-/-^ mice is lower than in NOD mice. Up: Expression in NOD.Rag2^-/-^ mice is higher than in NOD mice. Non-significant values are in red. The *asterisk* marks those values with a tendency to significance.

**Supplementary Table III. DEGs between mouse ages.**

| **Genes** | | **C57BL/6**  **3 vs. 12 weeks of age** | | | **NOD**  **3 vs. 12 weeks of age** | | | **NOD.RAG2^-/-^**  **3 vs. 12 weeks of age** | | |
| --- | --- | --- | --- | --- | --- | --- | --- | --- | --- | --- |
| **Symbol** | **Name** | **adjusted p-value** | **logFC** | **Up or Down** | **adjusted p-value** | **logFC** | **Up or Down** | **adjusted p-value** | **logFC** | **Up or Down** |
| *Abhd1* | *Abhydrolase domain containing 1* | 0,29 | -0,68 |  | 0,03 | 0,58 |  | 0,78 | 0,18 |  |
| *Ahcy* | *S-adenosyl-homocysteine hydrolase* | 0,85 | 0,23 |  | 0,21 | 0,58 |  | 0,95 | 0,09 |  |
| *Alad* | *Aminolevulinate, delta-, dehydratase* | 0,69 | 0,29 |  | 3,59E-03 | 1,08 | Down | 0,46 | 0,45 |  |
| *Arl8a* | *ADP-ribosylation factor-like 8A* | 0,93 | -0,08 |  | 0,58 | -0,17 |  | 0,76 | 0,23 |  |
| *Arntl2* | *Aryl hydrocarbon receptor nuclear translocator-like 2* | 0,60 | -0,31 |  | 0,79 | -0,07 |  | 0,68 | 0,24 |  |
| *Ccl19* | *Chemokine (C-C motif) ligand 19* | 0,67 | -0,33 |  | 0,01 | -0,78 | Up* | 0,87 | 0,21 |  |
| *Cisd3* | *CDGSH iron sulfur domain 3* | 0,97 | 0,03 |  | 0,12 | -0,39 |  | 0,55 | -0,33 |  |
| *Cst12* | *Cystatin 12* | 0,99 | 0,02 |  | 0,49 | -0,21 |  | 0,78 | -0,21 |  |
| *Fau* | *Finkel-Biskis-Reilly murine sarcoma virus (FBR-MuSV) ubiquitously expressed (fox derived)* | 0,89 | 0,10 |  | 0,52 | -0,17 |  | 0,67 | -0,25 |  |
| *Fbp2* | *Fructose biphosphatase 2* | 0,65 | 0,35 |  | 0,16 | -0,45 |  | 1,00 | 0,00 |  |
| *Gabra2* | *Gamma-aminobutyric acid (GABA) A receptor, subunit alpha 2* | 0,31 | -0,76 |  | 0,01 | -0,96 | Up* | 0,97 | -0,04 |  |
| *Gnpda1* | *Glucosamine-6-phosphate isomerase 1* | 0,93 | 0,10 |  | 0,04 | 0,64 |  | 0,47 | -0,54 |  |
| *Gsto1* | *glutathione S-transferase omega 1* | 0,60 | -0,33 |  | 0,61 | -0,14 |  | 0,64 | -0,28 |  |
| *H2afz* | *H2A histone family, member Z* | 0,80 | 0,24 |  | 0,61 | -0,20 |  | 0,49 | 0,54 |  |
| *H2-T22* | *Histocompatibility 2, T region locus 22* | 0,97 | 0,05 |  | 0,57 | 0,26 |  | 0,69 | -0,41 |  |
| *Hddc3* | *HD domain containing 3* | 1,00 | 0,00 |  | 0,22 | 0,42 |  | 0,65 | 0,36 |  |
| *Heatr5b* | *HEAT repeat containing 5B* | 0,69 | -0,27 |  | 0,59 | 0,16 |  | 0,64 | -0,31 |  |
| *Hist1h2b* | *H2B clustered histone 4* | 0,18 | 1,09 |  | 1,10E-03 | 1,50 | Down | 0,09 | 1,07 |  |
| *Hjurp* | *Holliday junction recognition protein* | 0,82 | 0,16 |  | 0,10 | 0,48 |  | 0,80 | 0,19 |  |
| *Hspe1* | *Heat shock protein l (chaperonin 10)* | 0,89 | -0,13 |  | 0,17 | 0,45 |  | 0,95 | -0,07 |  |
| *Ifna4* | *Interferon alpha 4* | 0,87 | 0,15 |  | 0,47 | -0,26 |  | 0,72 | 0,30 |  |
| *Itgb3bp* | *Integrin beta 3 binding protein (beta3-endonexin)* | 0,65 | -0,30 |  | 0,50 | 0,20 |  | 0,93 | 0,08 |  |
| *Lect1* | *Leucocyte cell derived chemotaxin 1* | 0,89 | -0,14 |  | 0,25 | 0,40 |  | 0,87 | 0,16 |  |
| *Ly86* | *Lymphocyte antigen 86* | 0,48 | -0,50 |  | 0,17 | -0,41 |  | 0,11 | -0,80 |  |
| *Mamdc2* | *MAM domain containing 2* | 0,86 | -0,15 |  | 0,27 | 0,36 |  | 0,89 | 0,14 |  |
| *Map7* | *Microtubule-associated protein 7* | 0,44 | -0,63 |  | 0,21 | -0,43 |  | 0,86 | -0,17 |  |
| *Mettl7a3* | *methyltransferase like 7A3* | 0,89 | -0,14 |  | 0,74 | -0,13 |  | 0,81 | -0,23 |  |
| *Mid1* | *Midline 1* | 0,73 | -0,24 |  | 0,92 | -0,03 |  | 0,42 | 0,47 |  |
| *Mmp25* | *Matrix metallopeptidase 25* | 0,48 | -0,48 |  | 0,11 | -0,46 |  | 0,88 | -0,13 |  |
| *Mrgpra2a/b* | *MAS-related GPR, member A2B* | 0,64 | -0,27 |  | 0,14 | -0,35 |  | 0,67 | -0,24 |  |
| *Mrgpra3-4* | *MAS-related GPR, member A3/A4* | 0,96 | 0,04 |  | 2,60E-03 | -1,03 | Up | 0,51 | -0,37 |  |
| *Mrgprb4* | *MAS-related GPR, member B4* | 0,18 | 1,08 |  | 0,66 | -0,17 |  | 0,90 | -0,13 |  |
| *Mrgprx1* | *MAS-related GPR, member X1* | 0,93 | -0,08 |  | 4,54E-04 | -1,34 | Up | 0,19 | -0,64 |  |
| *Myo7a* | *Myosin VIIA* | 0,57 | -0,37 |  | 0,43 | 0,22 |  | 0,49 | 0,37 |  |
| *Nts* | *Neurotensin* | 0,22 | -1,08 |  | 0,73 | 0,15 |  | 0,43 | -0,63 |  |
| *Nxpe2* | *Neurexophilin and PC-esterase domain family, member 2* | 0,84 | 0,15 |  | 0,26 | 0,34 |  | 0,38 | 0,51 |  |
| *Olfr1269* | *Olfactory receptor 1269* | 0,48 | 0,47 |  | 0,94 | -0,03 |  | 0,35 | -0,50 |  |
| *Osr2* | *Odd-skipped related 2* | 0,54 | -0,55 |  | 0,01 | 1,11 | Down | 0,30 | 0,71 |  |
| *Ostc* | *Oligosaccharyl transferase complex subunit* | 0,87 | 0,12 |  | 0,22 | 0,36 |  | 0,77 | 0,21 |  |
| *P4ha3* | *Procollagen-proline, 2-oxoglutarate 4-dioxygenase (proline 4-hydroxylase), alpha polypeptide III* | 0,51 | -0,46 |  | 0,83 | -0,07 |  | 0,98 | 0,02 |  |
| *Pianp* | *PILR alpha associated neural protein* | 0,66 | -0,29 |  | 0,67 | -0,13 |  | 0,98 | -0,02 |  |
| *Pisd-ps3* | *Phosphatidylserine decarboxylase, pseudogene 3* | 0,54 | -0,82 |  | 0,07 | -0,98 |  | 0,92 | 0,18 |  |
| *Pla2g4e* | *phospholipase A2, groDown IVE* | 0,77 | 0,17 |  | 0,14 | -0,36 |  | 0,97 | 0,03 |  |
| *Ppp1r3e* | *Protein phosphatase 1, regulatory (inhibitor) subunit 3E* | 0,63 | -0,32 |  | 0,96 | 0,02 |  | 0,89 | 0,12 |  |
| *Psmc3ip* | *Proteasome (prosome, macropain) 26S subunit, ATPase 3, interacting protein* | 0,97 | 0,04 |  | 0,51 | 0,22 |  | 0,94 | -0,08 |  |
| *Pttg1* | *Pituitary tumor-transforming gene 1* | 0,87 | -0,13 |  | 0,10 | 0,50 |  | 0,23 | 0,65 |  |
| *Rian* | *RNA imprinted and accumulated in nucleus* | 0,94 | -0,08 |  | 0,66 | -0,22 |  | 0,74 | 0,38 |  |
| *Rpl29* | *Ribosomal Protein L29* | 0,85 | -0,17 |  | 0,75 | 0,12 |  | 0,85 | -0,18 |  |
| *Rpl3* | *Ribosomal protein L3* | 0,95 | 0,11 |  | 0,26 | -0,64 |  | 0,31 | 1,07 |  |
| *Rpl32* | *Ribosomal protein L32* | 0,77 | 0,23 |  | 0,11 | -0,51 |  | 0,97 | 0,04 |  |
| *Rpl34* | *Ribosomal protein L34* | 0,82 | 0,25 |  | 0,27 | 0,50 |  | 0,46 | -0,65 |  |
| *Rps12* | *Ribosomal protein S12* | 0,89 | -0,16 |  | 0,02 | 0,95 | Down* | 0,68 | 0,40 |  |
| *Rps19-ps4* | *Ribosomal protein S19, pseudogene 4* | 0,38 | 0,53 |  | 0,02 | 0,64 |  | 0,99 | -0,01 |  |
| *Samd11* | *Sterile alpha motif domain containing 11* | 0,95 | 0,06 |  | 0,06 | -0,59 |  | 0,88 | -0,13 |  |
| *Scg5* | *Secretogranin V* | 0,63 | -0,28 |  | 0,06 | -0,45 |  | 0,80 | -0,16 |  |
| *Slc35a5* | *Solute carrier family 35, member A5* | 1,00 | 0,00 |  | 0,77 | 0,09 |  | 0,82 | -0,17 |  |
| *Slc39a8* | *Solute carrier family 39 (metal ion transporter), member 8* | 0,98 | 0,02 |  | 0,55 | -0,17 |  | 0,51 | -0,37 |  |
| *Slco1c1* | *Solute carrier organic anion transporter family, member 1c1* | 0,77 | -0,27 |  | 0,90 | -0,06 |  | 0,68 | 0,38 |  |
| *Srp54c* | *Signal recognition particle 54C* | 0,79 | -0,16 |  | 0,18 | 0,33 |  | 0,68 | 0,25 |  |
| *Tdg* | *Thymine DNA* | 0,94 | 0,09 |  | 0,07 | 0,72 |  | 0,90 | 0,15 |  |
| *Tgm5* | *Transglutaminase 5* | 0,77 | 0,21 |  | 0,02 | -0,76 |  | 0,96 | -0,05 |  |
| *Tgoln1-2* | *Trans-golgi network protein* | 0,92 | -0,07 |  | 0,05 | -0,45 |  | 0,80 | -0,15 |  |
| *Tmem132c* | *Transmembrane protein 132C* | 0,93 | -0,08 |  | 0,45 | -0,22 |  | 0,81 | 0,17 |  |
| *Tmem203* | *Transmembrane protein 203* | 0,67 | 0,21 |  | 0,65 | 0,11 |  | 0,82 | 0,13 |  |
| *Tmem254a/b/c* | *Transmembrane protein 254* | 0,75 | 0,18 |  | 0,18 | 0,32 |  | 0,57 | 0,29 |  |
| *Tmem40* | *Transmembrane protein 40* | 0,77 | 0,23 |  | 0,11 | -0,51 |  | 0,97 | 0,04 |  |
| *Tmem72* | *Transmembrane protein 72* | 0,95 | -0,06 |  | 0,03 | -0,60 |  | 0,95 | 0,05 |  |
| *Tpm3* | *Tropomyosin 3, gamma* | 0,81 | 0,17 |  | 0,30 | 0,30 |  | 0,59 | 0,35 |  |
| *Trpc3* | *Transient receptor potential cation channel, subfamily C, member 3* | 0,68 | -0,25 |  | 0,01 | -0,80 |  | 0,32 | -0,49 |  |
| *Vcp* | *Valosin containing protein* | 0,88 | 0,10 |  | 0,58 | 0,15 |  | 0,91 | -0,09 |  |
| *Zfp125* | *Zinc finger protein 125* | 0,82 | 0,32 |  | 0,91 | -0,07 |  | 1,00 | -0,01 |  |

Differences were considered significant when the adjusted p-value was less than 0.01 and the |LogFC| was greater than 1. Down: Expression in NOD.Rag2^-/-^ mice is lower than in NOD mice. Up: Expression in NOD.Rag2^-/-^ mice is higher than in NOD mice. Non-significant values are in red. The *asterisk* marks those values with a tendency to significance.
